# Supplementary figures and images for: Integrated Analysis of mRNAs and Long Non-Coding RNAs Expression of Oviduct That Provides Novel Insights into the Prolificacy Mechanism of Goat (Capra hircus)
Source: Genes (Basel). 2022 Jun 8;13(6):1031. doi: 10.3390/genes13061031 (PMC9222479; doi:10.3390/genes13061031)

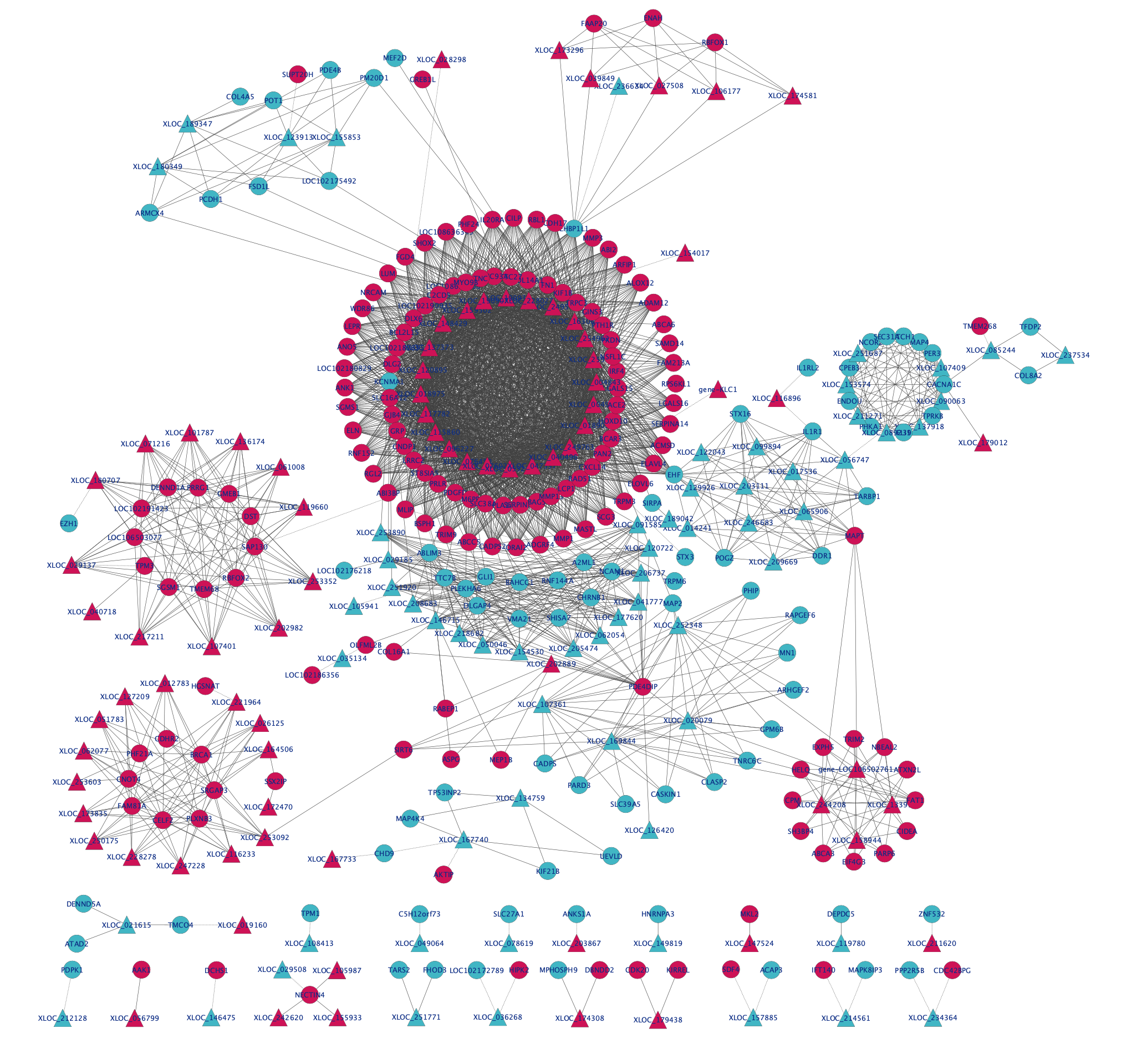

Supplement: Supplementary file 1 [file genes-13-01031-s001.zip › Figure S1.png]
